# Pilot trial results of D-HOMES: a behavioral-activation based intervention for diabetes medication adherence and psychological wellness among people who have been homeless

**DOI:** 10.3389/fpsyt.2024.1329138

**Published:** 2024-02-29

**Authors:** Katherine Diaz Vickery, Lillian Gelberg, Audrey Rose Hyson, Ella Strother, Jill Carter, Oscar Oranday Perez, Moncies Franco, Silvio Kavistan, Susan Gust, Edward Adair, Ali’Cia Anderson-Campbell, Lelis Brito, Annette Butler, Tahiti Robinson, John Connett, Michael D. Evans, Karen M. Emmons, W. Scott Comulada, Andrew M. Busch

**Affiliations:** ^1^The Health, Homelessness, and Criminal Justice Lab, Hennepin Healthcare Research Institute, Minneapolis, MN, United States; ^2^Department of Medicine, Hennepin Healthcare, Minneapolis, MN, United States; ^3^The Quorum for Community Engaged Wellness Research, Minneapolis, MN, United States; ^4^Department of Medicine, University of Minnesota, Minneapolis, MN, United States; ^5^David Geffen School of Medicine at UCLA, Los Angeles, CA, United States; ^6^UCLA Fielding School of Public Health, Los Angeles, CA, United States; ^7^The Behavioral Health Equity Research Group, Hennepin Healthcare Research Institute, Minneapolis, MN, United States; ^8^School of Public Health, University of Minnesota, Minneapolis, MN, United States; ^9^Clinical and Translational Science Institute, University of Minnesota, Minneapolis, MN, United States; ^10^Department of Social and Behavioral Sciences, Harvard T.H. Chan School of Public Health, Boston, MA, United States

**Keywords:** diabetes, health equity, homelessness, behavioral trials, behavioral activation

## Abstract

**Introduction:**

People living with type 2 diabetes who experience homelessness face a myriad of barriers to engaging in diabetes self-care behaviors that lead to premature complications and death. This is exacerbated by high rates of comorbid mental illness, substance use disorder, and other physical health problems. Despite strong evidence to support lay health coach and behavioral activation, little research has effectively engaged people living with type 2 diabetes who had experienced homelessness (DH).

**Methods:**

We used community engaged research and incremental behavioral treatment development to design the Diabetes HOmeless MEdication Support (D-HOMES) program, a one-on-one, 3 month, coaching intervention to improve medication adherence and psychological wellness for DH. We present results of our pilot randomized trial (with baseline, 3 mo., 6 mo. assessments) comparing D-HOMES to enhanced usual care (EUC; brief diabetes education session and routine care; NCT05258630). Participants were English-speaking adults with type 2 diabetes, current/recent (<24 mo.) homelessness, and an HbA1c‗7.5%. We focused on feasibility (recruitment, retention, engagement) and acceptability (Client Satisfaction Questionnaire, CSQ-8). Our primary clinical outcome was glycemic control (HbA1c) and primary behavioral outcome was medication adherence. Secondary outcomes included psychological wellness and diabetes self-care.

**Results:**

Thirty-six eligible participants enrolled, 18 in each arm. Most participants identified as Black males, had high rates of co-morbidities, and lived in subsidized housing. We retained 100% of participants at 3-months, and 94% at 6-months. Participants reported high satisfaction (mean CSQ-8 scores=28.64 [SD 3.94] of 32). HbA1c reduced to clinically significant levels in both groups, but we found no between group differences. Mean blood pressure improved more in D-HOMES than EUC between baseline and 6 mo. with between group mean differences of systolic -19.5 mmHg (*p*=0.030) and diastolic blood pressure -11.1 mmHg (*p*=0.049). We found no significant between group differences in other secondary outcomes.

**Conclusion:**

We effectively recruited and retained DH over 6 months. Data support that the D-HOMES intervention was acceptable and feasible. We observe preliminary blood pressure improvement favoring D-HOMES that were statistically and clinically significant. D-HOMES warrants testing in a fully powered trial which could inform future high quality behavioral trials to promote health equity.

**Clinical trial registration:**

https://clinicaltrials.gov/study/NCT05258630?term=D-HOMES&rank=1, identifier NCT05258630.

## 1 Introduction

Robust evidence ties social risks to persistent health inequities in premature morbidity and mortality due to diabetes (1). Homelessness is defined in the U.S. by the HEARTH Act and includes a dynamic spectrum including people staying in shelters, sleeping outside “or other places not meant for human habitation,” or who will imminently lose their housing (2). Homelessness is a key social risk that results in higher rates of diabetes-related complications and hospitalization (3, 4), and premature mortality compared to stably housed people (5). Homelessness presents substantial barriers to diabetes self-care, access to health care and prescription medications, managing psychological wellness and mental health, and affording and prioritizing diabetes care amidst other competing demands (6–8). Furthermore, homelessness has known association with premature morbidity and mortality (9), premature aging (10), and high rates of co-morbidities including trimorbidity, or the overlap of physical, mental health, and substance use conditions (11). Finally, the disproportionate impact of homelessness on people of color has deep roots in structural racism and may be a key driver of persistent racial/ethnic disparities in diabetes outcomes (12, 13).

Robust investment has yielded increasing evidence about how to deliver effective support to people living with diabetes (14, 15). A meta-analysis found that self-management education can reduce all-cause mortality in people with type 2 diabetes (16). Numerous one-on-one lay health coaching programs improve glycemic control in low resource environments (17–19). Strong evidence also supports approaches to co-manage diabetes and mental illness (20–22). While interventions to improve medication adherence across various diagnoses have not historically yielded conclusive evidence (23), growing literature suggests that behavioral activation techniques can effectively improve medication adherence and psychological wellness in populations facing resource constraints (24–26). Yet too often people living with type 2 diabetes who have experienced homelessness cannot access clinical trials or supportive programs for diabetes.

We sought to fill that gap by using community engaged research and incremental behavioral treatment development (27) to create a behavioral intervention tailored to the unique needs of people living with type 2 diabetes who have experienced homelessness. Guided by a team of people with lived experience and a treatment model based on the Information-Motivation-Behavioral Skills model (28), we conducted preliminary qualitative work with patients and housing and health care providers which revealed high desire and initial feasibility for a lay health coaching program focused on medication adherence and psychological wellness for people who had experienced homelessness with type 2 diabetes (29). Our community engaged research team, the Quorum for Community Engaged Wellness Research (Quorum), included people who had gained knowledge through lived experience of homelessness and diabetes, a community engaged research facilitator, and housing and health care providers. The Quorum guided all phases of this research to develop the Diabetes Homeless Medication Support (D-HOMES) program.

In this paper we present findings from our pilot randomized control trial comparing D-HOMES to enhanced usual care (EUC; one-time, brief diabetes education and encouragement to access existing clinical supports in our area). We report on feasibility, acceptability, and preliminary efficacy of clinical and behavioral outcomes.

## 2 Methods

### 2.1 Design

We conducted a two-arm, single blinded, randomized pilot trial comparing the feasibility, acceptability, and preliminary efficacy of D-HOMES versus EUC. We registered this trial at ClinicalTrials.gov (NCT05258630) and got approval by our Institutional Review Board. The first participant enrolled on February 23, 2022, and the last participant enrolled on January 27, 2023. While we could not blind coaches or participants, blinded staff collected all assessment data at baseline, 3-month, and 6-month time points. We conducted the study in an urban, Midwestern city in the United States. We compensated participants for each assessment visit ($150 total if all visits completed). We offered travel support with bus tokens, parking vouchers, or cab rides when needed. We also provided $20 per month to participants in both conditions who maintained a valid phone number to support cell phone charges. A participant who maintained a valid phone number could earn up to $120 across their 6 months in the study. We implemented this phone access incentive in response to feedback in previous studies indicating that many participants used “pay as you go” phones and/or had limited monthly cell phone minutes that were used up by study logistics. Note that we did not provide reimbursement for completing treatment sessions.

The Quorum team predates this trial and is ongoing currently. This team combines people with lived experience, researchers, and service providers in health care and housing. The team advised and monitored all stages of this trial. They impacted decisions including amount of compensation, presentation of informed consent using a clear infographic (29), the ethics of our comparison group, and reviewed all adverse events.

### 2.2 Participants

We set inclusion criteria of age 18 years or older, English-speaking, experience of homelessness in the past 24 months (per HEARTH Act) (2), self-reported diagnosis of type 2 diabetes verified in health record, HbA1c≥7.5% via study laboratory testing or clinical lab result in the last 30 days, plan to be reachable for the next 6 months, and willingness to work on medication adherence and diabetes self-care. Exclusion criteria were prior participation in earlier D-HOMES studies, inability to provide informed consent (e.g., presence of a legal guardian, active psychosis, or intoxication), and current pregnancy or lactation.

We attempted a variety of recruitment methods in this pilot trial. This included screening people with type 2 diabetes and evidence of homelessness in their medical records using a homelessness flag based on previous work by our team (30). We requested referrals from medical and behavioral health providers at area safety net clinics including Health Care for the Homeless, a national program offering clinical services to people experiencing homelessness (31). We also built partnerships with several local shelter and supportive housing providers for additional referrals, attempted outreach (e.g., tabling events), and we posted flyers in shelters, bus stops, drop-in centers, and local libraries.

We conducted telephone screening with interested participants who we invited to complete a two-part enrollment/baseline assessment process. The first visit included the informed consent process and collected initial demographic and related assessments and drew blood to confirm eligible HbA1c values. We invited for a second visit those with HbA1c values meeting inclusion criteria (or results completed in the last 30 days at a certified medical laboratory that we could see in electronic health records). During the second visit, participants completed remaining assessments. Participants then met with intervention coaches who completed randomization and initiated treatment conditions.

### 2.3 Randomization and intervention

A randomization scheme built into REDCap (32, 33) assigned participants to D-HOMES or enhanced usual care (EUC) using permutated block randomization. We randomized in a 1:1 ratio using small random sized blocks (with 2-4 participants per block).

#### 2.3.1 Diabetes homeless medication support

We detailed the development steps and treatment content of the Diabetes Homeless Medication Support (D-HOMES) program in a separate publication including our treatment model (29). Briefly, D-HOMES coaches used behavioral activation (BA) to work with participants to set weekly, personalized goals to improve medication adherence and psychological wellness (34). They offered participants in-person and telephone meetings for approximately 30 minutes weekly for up to 10 sessions from month 0-3 and up to 3 monthly 10-15 minute booster calls from months 4-6. Throughout treatment, coaches provided diabetes education on the topics and in formats desired by the participants and encouraged them to have and use a regular source of health care to support diabetes management, maintain prescriptions, and address any medication side effects. Initial sessions used a values exercise to identify sources of meaning and motivation specific to each participant. Coaches also explored potential connections between the participant’s values and diabetes management and returned to these connections throughout coaching to support motivation for diabetes management. Goal setting started in session 2 and focused on blood sugar control goals (mostly diabetes pill medication adherence, but also adherence to injectable medications including insulin, recommended blood sugar checks, and the interaction of diet and physical activity with blood sugar) and psychological wellness. For most participants, working on adherence to daily diabetes pill medication overlapped with daily adherence to other medications (e.g., blood pressure, cholesterol, and mental health). At sessions 3-5, coaches worked with each participant to choose one personalized commercially available tool (approximately $20 value) to support medication adherence, e.g.) pill organizer, notebook to record blood sugar levels, zipped pouch to store medications and glucometer. Booster calls reinforced goal-setting focused on reinforcing diabetes medication adherence. Throughout treatment, coaches provided referrals to area Health Care for the Homeless clinics and other homelessness and behavioral health services as needed.

#### 2.3.2 Enhanced usual care

Following the Pragmatic Model for Comparator Selection in Health-Related Behavioral Trials and with input from the Quorum team, we designed an enhanced usual care (EUC) comparator. This aligned with our study’s purpose, the phase of our research, and the real-world context and ethical issues of diabetes care for people who have experienced homelessness (35).

In EUC, the same coaches (OOP, JC) delivered one brief (approximately 15 minute), instructional diabetes education session to participants. They read the content of 3 handouts focused on (i) what is type 2 diabetes, (ii) healthy eating, and (iii) physical activity when living with diabetes used in a previous trial of people with diabetes from a low-income, urban area in the U.S (36–38). They also encouraged participants to use a regular source of health care to support diabetes management and supported participants to access such care if not already present. Coaches provided a resource page with area medical and behavioral health providers and homelessness resources to all participants.

#### 2.3.3 Interventionist training and fidelity monitoring

Coaches (OOP, JC) trained for approximately 22 hours in BA, motivational interviewing, homelessness and expected comorbidities, as well as diabetes. Each coach completed self-rated fidelity checklists after each visit in both arms. To establish supervisor rated fidelity, the PI (KDV) reviewed 35% of audio recordings of D-HOMES treatment sessions and completed the same session checklist. Weekly supervision meetings (led by KDV and AMB) supported coaching and ensured timely re-training for any departures from the protocol or treatment manual.

### 2.4 Feasibility and acceptability

Given the lack of prior longitudinal behavioral trials in this population, we primarily sought to establish feasibility and acceptability in this pilot trial. We measured feasibility by tracking the recruitment and retention rates demonstrating the study team’s ability to connect with participants and follow the trial protocol. We measured acceptability with treatment engagement measures and the 8-item Client Satisfaction Questionnaire (CSQ-8). Each item is scored on a 4-point Likert like scale resulting in a range of scores from 8-32 higher scores indicating higher satisfaction (39, 40). We also collected fidelity data about the team’s ability to follow the treatment manual and study protocol (See 2.3.3).

### 2.5 Outcomes

Note that we labeled HbA1c as our “primary clinical outcome” because it is the primary outcome of interest in this line of work, however, this clinical trial is not powered for HbA1c. Likewise, we label medication adherence as our “primary behavioral target,” but we do not have power to detect differences in medication adherence. Thus, outcome results on our primary (and secondary) outcomes should be considered preliminary.

#### 2.5.1 Primary clinical outcome

Our primary clinical outcome was glycemic control measured by hemoglobin A1c (HbA1c). This widely used measure provides an estimated average glucose level across the past 3 months (41). HbA1c over 6.5% diagnoses diabetes (42) and clinical guidelines recommend each patient set an individualized goal with their health care provider based on a variety of factors (43). A change of 0.5% in HbA1c is considered clinically meaningful (44). We began measuring HbA1c using fingerstick samples from participants collected by research staff and transitioned to using serum samples collected in a certified medical lab due to multiple clotted specimens and participant preference for venipuncture. Our fingerstick specimens were processed using the DCA Vantage point-of-care machines (45); our venipuncture specimens were processed using the Sebia Capillarys Flex analyzer (46).

#### 2.5.2 Primary behavioral outcome

We found no research to inform the appropriateness of self-report medication adherence scales in this population, thus we collected self-reported medication adherence in multiple ways in this pilot to inform our choice of instrument for a future, larger trial. We measured medication adherence specific to diabetes using the Adherence to Refills and Medicines Scale for Diabetes, on which scores range from 11-44, with higher scores indicating more problems with adherence [ARMS-D; Mayberry et al., (47)]. We also used the glucose subscale of the Diabetes Self-Management Questionnaire (DSMQ) where total and subscale scores are transformed onto a 0 to 10 scale with higher scores indicating better self-management behaviors. A “not applicable” option allows some items to be excluded from the scale, e.g., for people who are not prescribed blood glucose checks (48). We measured overall medication adherence using the 12-item Adherence Starts with Knowledge (ASK-12) scale which allows a score range of 12-60 with higher scores indicating more barriers to adherence (49). We included an extra question from the 20-item ASK scale that uses a Likert-like agreement scale about alcohol use interfering with medication adherence (50). We adapted our own question about use of drugs (“My use of drugs gets in the way of taking my medicines.”). We report on results of these added alcohol and drug interference questions separately from the standard scales.

#### 2.5.3 Secondary outcomes

We measured psychological wellness with the 5-item Mental Health Inventory (MHI-5), a brief transdiagnostic screening tool attuned to broader concepts of wellbeing and distress than other available measures. MHI-5 scores are computed and transformed on a 0 to 100 point scale with higher scores indicating more wellness (51). We also included the 5-item Problem Areas in Diabetes (PAID-5) scale which results in scores from 0 to 20 with higher scores indicating more distress (52). Finally, we collected the diet, physical activity, and health care use subscales of the Diabetes Self-Management Questionnaire [DSMQ; Schmitt et al., (48)]. The DSMQ is an 16-item instrument which asks questions about a variety of behaviors related to diabetes self-management. We found it to be more appropriate for our population than other commonly used measures which reference employment and things done “around the house” (53).

Given the known importance of weight and blood pressure on long-term outcomes of people living with type 2 diabetes, we also measured Body Mass Index (BMI, weight divided by height squared) and blood pressure using standard instruments and protocols (54).

Given prior work by our team and others documenting high rates of substance use among people experiencing homelessness (11, 55, 56), we used an adapted version of the ASSIST tool to document types and frequency of use of tobacco, alcohol, and other drugs (57).

### 2.6 Statistical analysis

We planned an intention-to-treat approach and prespecified our statistical endpoints while recognizing we are not recruiting a sample size large enough to be fully powered on any outcome in this pilot trial. We set a two-tailed significance level (alpha) of 5% for our findings. We used R to summarize REDCap data extracts for presentation and calculate scores according to published literature. We used mixed-effects linear models with fixed effects terms for intervention, assessment time point, and intervention-by-time point interaction, and a random effect term for participant to account for within-participant correlation across visits. We examined the treatment effect by testing the interaction terms for the 3- and 6-month visits with baseline visit treated as the reference level. Linear mixed models provide unbiased estimation of the treatment effect in the presence of missing outcome data under the missing random assumption. We summarize results using means with 95% confidence intervals and p-values from these models. Analyses were conducted using R version 4.2.2 (58).

## 3 Results

### 3.1 Feasibility and acceptability outcomes and sample characteristics

**Figure 1:**
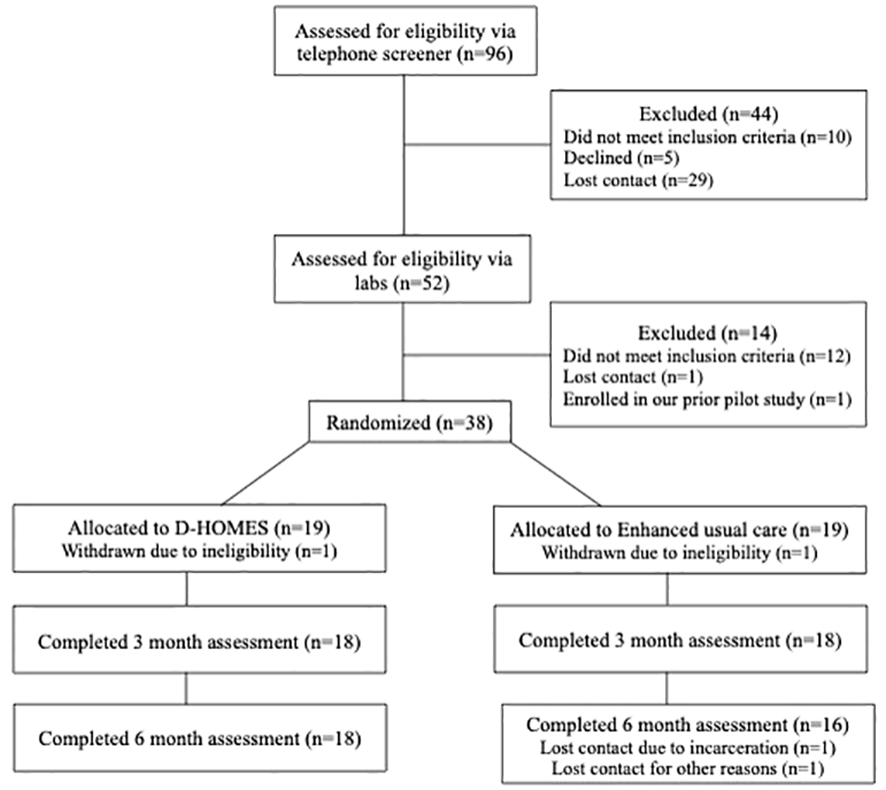
Recruitment and retention of participants in the D-HOMES pilot trial.

From February 23, 2022 to January 27, 2023 we initiated screening with 96 people, fully screened 52 participants (4.7 per month) and enrolled 36 eligible participants (3.3 per month). We found screening people with upcoming medical visits with evidence of diabetes and homelessness in their medical records to be our most effective recruitment strategy (n = 24). Letters to people with diabetes diagnosis and evidence of homelessness (n=6); flyers posted in homeless shelters, service centers, public libraries, and bus stops (n = 3); and direct referrals from housing (n=2) and medical providers (n=1) also resulted in some eligible participants. Outreach (tabling) efforts at homeless drop-in centers and housing facilities did not result in any eligible participants. At the 3 month assessment 100% of eligible participants provided primary clinical outcome data (HbA1c), and 94% provided this data at the 6 month assessment (**Figure 1**).

The majority of our final eligible sample of 36 participants identified as Black, non-Hispanic males with a mean age 52 years and were prescribed a mean of approximately 6 medications each (**Table 1**). Homeless experiences varied with most participants currently living in subsidized housing (transitional or supportive) or staying at homeless shelters for the majority of the 30 days prior to enrollment. Participants reported high mean counts of lifetime homeless episodes (D-HOMES 4.2 [SD 5.6], EUC 2.4 [SD 1.7]). Participants reported high rates of co-morbidities with high blood pressure, high cholesterol, depression, anxiety/panic disorder, and post-traumatic stress disorder being the highest reported comorbidities. They also had high rates of traumatic brain injury, bipolar disorder, and schizophrenia/schizoaffective disorder. Participants reported high rates of substance use especially tobacco and alcohol, and 2 participants reported prior lifetime overdoses.

**Table 1:** Demographic, social, and medical characteristics of pilot trial participants.

n (%) unless otherwise specified	D-HOMES	EUC
**N**	18	18
**Age** (mean years, SD)	52.2 (10.2)	51.9 (10.8)
Gender^@^		
Female	5 (27.8)	6 (33.3)
Male	13 (72.2)	12 (66.7)
Race^		
Black	16 (88.9)	13 (72.2)
White	1 (5.6)	2 (11.1)
Other	2 (11.1)	3 (16.7)
American Indian	1 (5.6)	1 (5.6)
Native Hawaiian or Pacific Islander	1 (5.6)	0
**Hispanic Ethnicity** (yes)	1 (5.6)	1 (5.6)
**Number of prescribed medications**^#^ (mean, SD)	5.9 (2.5)	6.4 (2.1)
Education		
Less than high school	6 (33.3)	6 (33.3)
High school graduate/GED	6 (33.3)	3 (16.7)
Some college/technical degree/Associate degree	6 (33.3)	8 (44.4)
College graduate (BA or BS)	0 (0.0)	1 (5.6)
Health insurance		
Insurance through exchanges	2 (11.1)	1 (5.6)
Medicaid or MinnesotaCare	9 (50.0)	7 (41.2)
Medicare	3 (16.7)	2 (11.8)
Other	4 (22.2)	6 (35.3)
Don’t know	0	1 (5.6)
Missing	0	1 (5.6)
Housing at enrollment, majority in the past 30 days		
Homeless shelter	5 (27.8)	2 (11.1)
Someone else’s apartment/room/house	1 (5.6)	1 (5.9)
Own apartment/room/house with subsidy	7 (38.9)	7 (38.9)
Own apartment/room/house without subsidy	1 (5.6)	6 (35.3)
Halfway house, residential treatment (drug/alcohol) program	1 (5.6)	0
Transitional housing	1 (5.6)	1 (5.6)
Other	2 (11.1)	0
Missing	0	1 (5.6)
**Count of lifetime homeless episodes**,* 30+ days (mean, SD)	4.2 (5.6)	2.4 (1.7)
Co-morbidities,^ told by health care professional		
High blood pressure	9 (50.0)	12 (66.7)
High cholesterol	11 (61.1)	10 (55.6)
Depression	10 (55.6)	9 (50.0)
Anxiety/panic disorder	7 (38.9)	9 (50.0)
Post-traumatic stress disorder (PTSD)	6 (33.3)	7 (38.9)
Arthritis	5 (27.8)	7 (38.9)
Asthma	7 (38.9)	3 (16.7)
Emphysema or COPD or chronic bronchitis	2 (11.1)	4 (22.2)
Heart disease	1 (5.6)	2 (11.1)
Liver problems	0	1 (5.6)
Traumatic Brain Injury (TBI)	1 (5.6)	0
Bipolar disorder	6 (33.3)	2 (11.1)
Schizophrenia/Schizoaffective	5 (27.8)	1 (5.6)
None	1 (5.6)	1 (5.6)
Reported substance use,^ any in past 3 months		
Tobacco^#^	8 (44.4)	11 (64.7)
Alcohol	7 (38.9)	13 (72.2)
Cannabis^#^	5 (27.8)	7 (41.2)
Cocaine^&^	2 (11.1)	3 (18.8)
Amphetamine	2 (11.1)	0
Inhalant	0 (0.0)	1 (5.6)
Sedatives	6 (33.3)	3 (16.7)
Opioids	1 (5.6)	1 (5.6)
**History of overdose**	0	2 (11.1)

^@^“Non-binary or other” response also offered, not reported by any participants.

^Multiple answers per participant allowed for this item.

^#^1 participant with missing data.

^&^2 participants with missing data.

*3 participants with missing data.

Participants demonstrated high treatment engagement in the D-HOMES intervention, suggesting good acceptability. D-HOMES participants completed an average of 8.7 coaching sessions (of 10 offered). Most participants randomized to D-HOMES (n=13/18) received all 10 sessions. Participants set an average of 2.5 goals/session and reported completing 73.8% of goals set. D-HOMES participants reported a mean score of 29.06 (SD 4.26, range 17, 32) on the Client Satisfaction Questionnaire indicating high satisfaction with the D-HOMES intervention. Those in the EUC condition reported a mean Client Satisfaction Questionnaire score of 28.22 (SD 3.66, range 22, 32) indicating high satisfaction with the EUC education intervention.

Fidelity checks revealed that coaches followed the treatment manual and study protocol for D-HOMES and EUC arms. EUC self-ratings revealed 100% fidelity to the protocol. D-HOMES checklists demonstrated high fidelity in both self-ratings (97.8%) and supervisor ratings (96.9%).

### 3.2 Outcome analyses

#### 3.2.1 Primary clinical outcome

**Figure 2:**
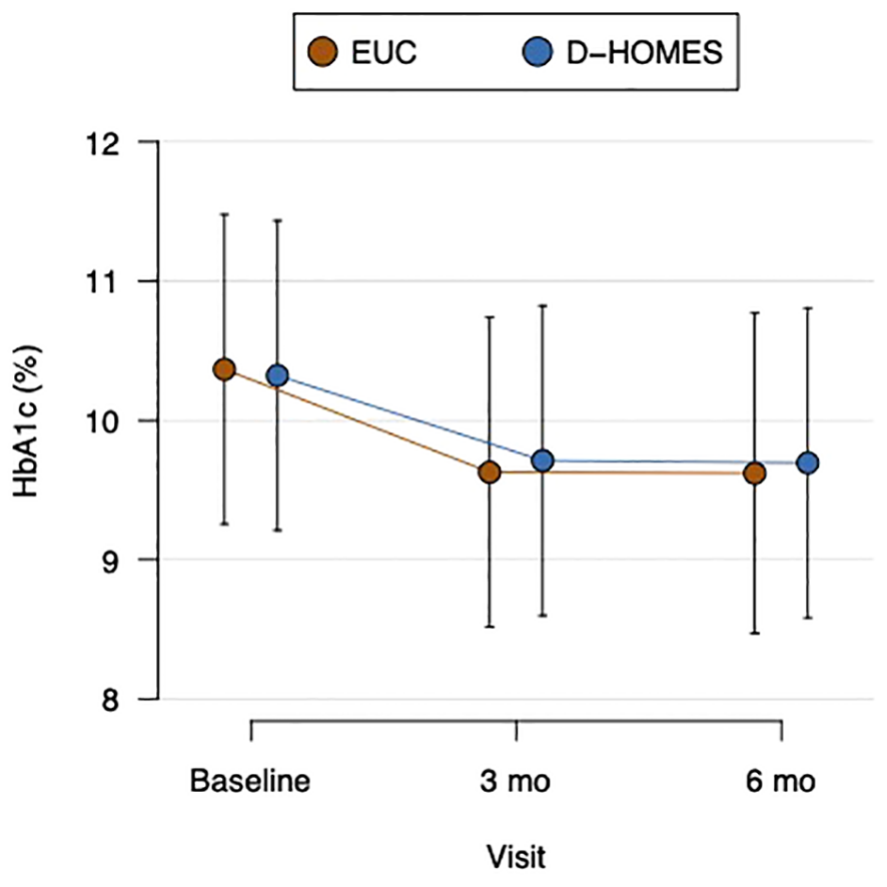
Change in glycemic control (HbA1c) in D-HOMES and enhanced usual care.

We found no between group differences in mean HbA1c change over time (**Figure 2**; **Table 2**). We note that both D-HOMES and EUC participants experienced a clinically significant (>0.5%) reduction in mean HbA1c from baseline to 3 months (D-HOMES -0.6% [SD 2.4%], EUC -0.8% [SD 2.5%]) and maintained that reduction through 6 months (baseline to 6 months mean change: D-HOMES -0.6% [SD 2.7%], EUC -0.8% [SD 2.5%]).

**Table 2:** Medical and psychological outcome differences over time between D-HOMES and enhanced usual care.

	Baseline, mean (SD)		3-months, mean (SD)		Change baseline to 3-months	6-months, mean (SD)		Change baseline to 6-months
	EUC	D-HOMES	EUC	D-HOMES	DH vs. EUC (95% CI), *d*, p-value	EUC	D-HOMES	DH vs. EUC. (95% CI), *d*, p-value
Primary clinical outcome								
HbA1c (%)	10.4 (2.2)	10.3 (2.1)	9.6 (2.4)	9.7 (2.3)	0.1 (-1.3, 1.5), *d*=0.05, p=0.861	9.6 (2.4)	9.7 (2.7)	0.1 (-1.3, 1.6), *d*=0.05, p=0.873
Primary behavioral outcome (medication adherence)								
Diabetes medication adherence (ARMS-D)	17.2 (3.9)	15.6 (4.6)	16.7 (3.4)	16.0 (5.2)	0.9 (-1.2, 3.1), *d*=0.26, p=0.400	16.1 (3.7)	15.2 (5.2)	0.6 (-1.6, 2.8), *d*=0.24, p=0.583
Diabetes self-management (DSMQ), Glucose scale	6.6 (3.0)	6.7 (2.5)	6.9 (2.2)	8.1 (2.4)	1.0 (-0.3, 2.4), *d*=0.45, p=0.148	7.1 (2.7)	8.3 (2.2)	1.0 (-0.4, 2.4), *d*=0.43, p=0.179
All medication adherence (ASK-12)	32.9 (7.7)	36.3 (8.5)	35.2 (5.6)	36.9 (6.6)	-1.6 (-6.4, 3.2), *d*=-0.20, p=0.521	32.1 (8.5)	36.4 (6.2)	1.0 (-4.0, 5.9), *d*=0.12, p=0.709
Drug/alcohol use impacting medication adherence (% yes)	22.2	16.7	16.7	5.6	-5.6 (-32.9, 21.8), *d*=NA, p=0.696	14.2	11.1	2.5 (-25.3, 30.4), *d*=NA, p=0.865
Secondary outcomes								
Psychological wellness (MHI-5)	39.8 (21.2)	34.4 (24.9)	37.6 (17.3)	38.0 (20.2)	5.8 (-6.9, 18.4), *d*=0.34, p=0.382	36.7 (24.4)	34.7 (20.6)	3.3 (-9.7, 16.2), *d*=0.15, p=0.629
Diabetes distress (PAID-5)	7.6 (7.2)	8.2 (6.5)	8.1 (7.0)	7.2 (5.8)	-1.5 (-4.1, 1.1), *d*=-0.42, p=0.269	6.9 (6.6)	5.8 (5.4)	-1.7 (-4.4, 0.9), *d*=-0.39, p=0.219
DSMQ, overall	4.7 (1.2)	5.1 (1.2)	5.0 (1.0)	5.6 (1.2)	0.2 (-0.5, 1.0), *d*=0.17, p=0.591	5.1 (1.0)	5.2 (1.1)	-0.3 (-1.0, 0.5), *d*=-0.21, p=0.509
DSMQ Diet scale	4.9 (2.5)	5.4 (2.2)	5.0 (2.4)	6.0 (2.2)	0.4 (-1.2, 1.9), *d*=0.17, p=0.642	5.8 (2.1)	5.9 (2.2)	-0.4 (-2.0, 1.1), *d*=-0.18, p=0.583
DSMQ Physical activ. scale	3.6 (2.3)	3.9 (2.9)	3.5 (2.6)	4.0 (2.9)	0.3 (-1.5, 2.2), *d*=0.10, p=0.749	3.1 (1.8)	2.6 (2.5)	-0.8 (-2.7, 1.1), *d*=-0.28, p=0.418
DSMQ Health care use scale	2.0 (2.1)	2.3 (2.2)	2.7 (2.6)	2.1 (1.9)	-0.9 (-2.5, 0.8), *d*=-0.32, p=0.307	1.7 (1.7)	1.4 (1.7)	-0.6 (-2.3, 1.0), *d*=-0.25, p=0.467
BMI	36.8 (12.8)	31.6 (7.0)	34.7 (10.4)	31.4 (7.2)	2.0 (-0.6, 4.6), *d*=0.40, p=0.144	36.4 (11.9)	31.0 (6.6)	-0.2 (-2.8, 2.5), *d*=-0.08, p=0.906
Systolic blood pressure (mmHg)	131.6 (17.1)	138.1 (19.8)	139.0 (23.4)	132.9 (19.0)	-12.7 (-29.3, 4.0), *d*=-0.55, p=0.147	139.1 (23.4)	126.2 (22.0)	-19.5 (-36.5, -2.6), *d*=-0.69, p=0.030**
Diastolic blood pressure (mmHg)	82.3 (10.2)	84.1 (11.9)	89.1 (15.5)	83.4 (9.4)	-7.5 (-18.0, 3.0), *d*=-0.49, p=0.175	86.8 (16.1)	77.5 (8.9)	-11.1 (-21.9, -0.4), *d*=-0.57, p=0.049**

** Change meeting pre-determined significance threshold of p<.05.

#### 3.2.2 Primary behavioral outcome

We observed a skewed score distribution on our measure of diabetes specific medication adherence (ARMS-D) at baseline with significant ceiling effects (**Table 2**). The mode ARMS-D score at baseline was 11 (n = 7; 22% of sample), meaning no self-reported problems with adherence, and 18 participants (50% of sample) reported a score of 15 or less (rare adherence problems) at baseline. This allowed for little improvement over time. We did not observe any significant between group effects over time on the ARMS-D.

The glucose subscale of the DSMQ demonstrated a better array of scores (**Table 2**) with fewer ceiling effects. Glucose subscale scores improved somewhat in both groups, although we saw slightly more mean change in the D-HOMES vs. EUC (baseline to 3 mo. between group difference in change: 1.0 [95% CI -0.3, 2.4], baseline to 6 mo. between group difference in change: 1.0 [95% CI -0.4, 2.4]).

Overall medication adherence as measured by ASK-12 total scores demonstrated little change across time points among our participants. A significant minority of participants (16.7% in D-HOMES and 22.2% in EUC) reported that drugs and/or alcohol interfered with medication adherence; however, we found no significant between group mean differences in drug/alcohol interference over time.

#### 3.2.3 Secondary outcomes

We report all secondary outcome data in **Table 2**. Participants demonstrated low levels of psychological wellness (MHI-5) and high levels of diabetes distress (PAID-5), but there were no significant between group differences on either scale. The remaining 3 DSMQ subscales showed no significant between group differences. Mean BMI was in the obese range (30–34, 36–41) for both D-HOMES (31.6) and EUC (36.8) groups at baseline, and BMI showed no significant between group differences over time.

**Figure 3:**
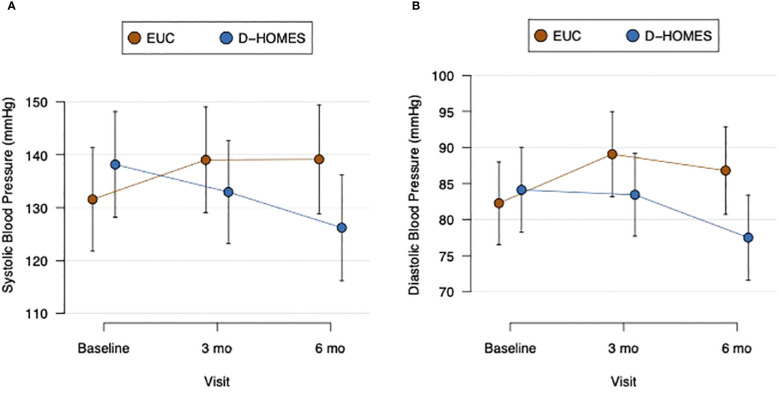
Change in systolic **(A)** and diastolic **(B)** blood pressure in D-HOMES and enhanced usual care.

Mean blood pressure at baseline was 138.1/84.1 among D-HOMES participants and 131.6/82.3 among EUC participants (**Table 2**). We observed a significantly larger reduction in systolic blood pressure from baseline to 6 months in D-HOMES (-11.9mmHg) vs EUC (+7.5mmHg); (**Figure 3**; **Table 2**). We observed a significantly larger reduction in diastolic blood pressure from baseline to 6 months in D-HOMES (-6.6 mmHg [SD 15.7]) vs EUC (+4.5 mmHg [SD 21.6]) (**Figure 3**; **Table 2**).

### 3.3 Adverse events

We actively tracked adverse events throughout this trial and reviewed them per protocol. One participant had an amputation during the study, which we reported to the IRB who deemed this a serious adverse event unrelated to the study’s activities. No other adverse events occurred.

## 4 Discussion

We developed a randomized trial protocol to compare the D-HOMES intervention to Enhanced Usual Care (EUC). D-HOMES is a behavioral program tailored to the unique needs people living with type 2 diabetes who had experienced homelessness (DH) developed via an incremental, community engaged behavioral trial development process. We found D-HOMES and our trial protocol feasible and acceptable to DH with both current or recent homelessness. We found that reaching out to patients already engaged in care at medical clinics best supported recruitment. High staff turnover and open positions in housing and homeless-focused health care settings due to the COVID-19 pandemic limited referrals from these locations and challenged our recruitment. This led us to close enrollment before reaching our initial planned target (n=54) and may limit the generalizability of our pilot trial results. We attribute our overall high retention to the careful creation of a protocol with input from the Quorum team that incentivized participants to remain in contact with the study team each month. We found $20/month to be an appropriate and effective telephone stipend that our target population found meaningful.

Our study adds to emerging models in the literature of innovations and clinical trials to address type 2 diabetes among people experiencing homelessness (6, 59, 60). However, our work is novel in our focus on a high-quality behavioral intervention with a treatment manual, fidelity measurement, and an incremental treatment development approach. We are also novel in our collaboration with a community engaged research team (Quorum) who has guided this work from conceptualization through pilot trial results. The team collaborated on designing a fully powered hybrid trial, currently under review, as a planned next step in our work. We will also consider further adaptions to better serve people staying outside, or in other places not meant for human habitation, as we did not reach this segment of the homeless population in our work to date.

In this feasibility-focused pilot trial, we were underpowered to detect between group differences in our primary clinical outcome of glycemic control (HbA1c). While we found no between group effect, we were encouraged to find a clinically significant (≥0.5%) improvement in glycemic control in both groups. This may reflect the impact among EUC participants of even one-time brief education and resource support as well as regular contact with our study staff and receipt of $20/month. We continue make improvements to the D-HOMES manual to intensify the treatment and maximize efficacy. For example, we are presently developing augmented educational content for D-HOMES enrollees with input from the Quorum team. We also plan to add an offer of continuous glucose monitoring to all D-HOMES participants during the initial coaching session to enhance the self-monitoring activity (61). We are also planning to add a longer, in-person second coaching session to review medications and diabetes supplies in participants’ homes or natural environments (subsidized apartments, vehicles, shelters, etc.).

While this study was not designed for scale validation, given the dearth of relevant literature, our results can help inform future choices regarding measurement of self-reported diabetes medication adherence among DH. The ARMS-D scale showed substantial ceiling effects and did not improve over time as would be expected given the observed improvement in HbA1c over time. This deviates from observed patterns in other adults with type 2 diabetes (47). The glucose subscale of the DSMQ showed a better distribution and did improve concurrently with HbA1c over time as has been seen in other studies of adults with diabetes (62).

Our challenges using self-report adherence measures parallel struggles other researchers have encountered but may have unique implications in the DH population (63). Participants may have specifically struggled to summarize their adherence to diabetes regimens combining oral and injectable medications and glucose monitoring schedules. They may also have been unaware of their fully prescribed regimen especially at baseline. Specific barriers to accurate self-report may also arise from the complex lives of DH participants who face many competing demands for their time and attention. Participants shared numerous psychosocial stressors with the study team including frequently lost phones, medications, and other belongings; frequent relocation; and being impacted by violence towards their friends and family members. These findings reinforce our plans to focus on glycemic control (HbA1c) as the primary outcome of a larger, fully powered trial.

Psychological wellness (MHI-5) demonstrated scores that indicate majority of participants would meet one suggested diagnostic cut point for mental illness (<76) at all study time points (64). This, along with self-reported comorbidity diagnoses and substance use (**Table 1**), confirm that our study population aligns with other profiles in the literature of adults who experience homelessness (9, 11). The overlap of race/ethnicity, multiple comorbidities, and social inequities imposed by ongoing structural racism and discrimination within housing and health care systems align with the intersectionality literature which highlights the “multiple burdens” facing some populations that perpetuate social inequality (65, 66). They also connect directly to constructs of structural vulnerability, or the pathways of power relationships that exacerbate health problems. Structural vulnerabilities have been connected to the need for multi-disciplinary health and social services (67). With the continued refinement of D-HOMES, we look forward to continued study of how a behavioral treatment can target such complex needs and how psychological wellness can best be measured in such a context.

We observed a clinically meaningful and sustained reduction in both systolic and diastolic blood pressure among D-HOMES participants at 6-months. This is particularly important because the mean blood pressure among D-HOMES participants at the 6-month point met current guidelines recommended by the American Diabetes Association (<130/80) (68). Guidelines focus on the strong evidence connecting hypertension as a risk factor for development of atherosclerotic cardiovascular disease, heart failure, and microvascular diabetes complications for people living with diabetes (69).

Overall, we conclude that it is possible to recruit and retain people living with diabetes who have experienced homelessness when community engaged research approaches are used to align study protocols to the needs of participants (e.g., providing monthly phone payments). D-HOMES warrants testing in a fully powered trial. With such testing D-HOMES could inform future efforts to use high quality behavioral trials to promote health equity for people facing the severe social risk of homelessness.

## Data availability statement

The datasets presented in this study can be found in online repositories. The names of the repository/repositories and accession number(s) can be found below: This trial is under review at clincialtrials.gov, NCT05258630.

## Ethics statement

The studies involving humans were approved by Hennepin Healthcare Research Institute. The studies were conducted in accordance with the local legislation and institutional requirements. The participants provided their written informed consent to participate in this study.

## Author contributions

KV: Conceptualization, Funding acquisition, Investigation, Supervision, Writing – original draft, Writing – review & editing. LG: Conceptualization, Funding acquisition, Investigation, Supervision, Writing – review & editing. AH: Conceptualization, Investigation, Project administration, Supervision, Writing – review & editing. ES: Data curation, Investigation, Project administration, Writing – review & editing. JiC: Investigation, Methodology, Writing – review & editing. OO: Investigation, Methodology, Writing – review & editing. MF: Project administration, Resources, Writing – review & editing. SK: Data curation, Investigation, Project administration, Writing – review & editing. SG: Conceptualization, Resources, Writing – review & editing. EA: Conceptualization, Resources, Writing – review & editing. AA: Conceptualization, Resources, Writing – review & editing. LB: Investigation, Resources, Writing – review & editing. AB: Investigation, Resources, Writing – review & editing. TR: Investigation, Resources, Writing – review & editing. JoC: Conceptualization, Funding acquisition, Investigation, Methodology, Supervision, Writing – review & editing. ME: Data curation, Formal analysis, Investigation, Methodology, Validation, Writing – review & editing. KE: Methodology, Supervision, Writing – review & editing. WC: Methodology, Supervision, Writing – review & editing. AB: Conceptualization, Funding acquisition, Investigation, Methodology, Supervision, Writing – review & editing.
